# *NOCTURNIN* Gene Diurnal Variation in Healthy Volunteers and Expression Levels in Shift Workers

**DOI:** 10.1155/2019/7582734

**Published:** 2019-07-31

**Authors:** Massimo Bracci, Alfredo Copertaro, Veronica Ciarapica, Mariella Barbaresi, Stefano Esposito, Antonella Albanesi, Matteo Valentino, Caterina Ledda, Venerando Rapisarda, Lory Santarelli

**Affiliations:** ^1^Occupational Medicine, Department of Clinical and Molecular Sciences, Polytechnic University of Marche, Ancona, Italy; ^2^Healthcare Workers Service, ASUR Area 2, Loreto Hospital, Loreto, Italy; ^3^Nutrition Service, ASUR Area 2, Loreto Hospital, Loreto, Italy; ^4^Occupational Medicine, Department of Clinical and Experimental Medicine, University of Catania, Catania, Italy

## Abstract

**Objective:**

The* NOCTURNIN* gene links nutrient absorption and metabolism to the circadian clock. Shift workers are at a heightened risk of overweight and of developing obesity and metabolic syndrome. This study investigates the diurnal variation of* NOCTURNIN* in healthy volunteers and its expression levels in rotational shift and daytime workers.

**Methods:**

* NOCTURNIN* expression levels were evaluated in peripheral blood lymphocytes from 15 healthy volunteers at 4-hour intervals for 24 h. Metabolic parameters and* NOCTURNIN* expression were measured in workers engaged in shift and daytime work.

**Results:**

In the group of volunteers* NOCTURNIN* expression showed diurnal variation, with a peak at 8:00 AM.* NOCTURNIN* expression was higher in shift workers than in daytime workers. Multivariate analysis confirmed the role of shift work as an independent factor affecting* NOCTURNIN* expression. Notably, its level correlated directly with body mass index and inversely with total energy expenditure.

**Conclusions:**

Measuring* NOCTURNIN* expression levels in human peripheral blood lymphocytes can improve investigations on the relationship between changes in circadian rhythm and metabolic disorders. Shift workers show higher* NOCTURNIN* levels than daytime workers.

## 1. Introduction

The* NOCTURNIN* gene (also known as* NOCT, NOC, CCR4L, Ccr4c*, and* CCRN4L*) was identified by Green et al. in 1996 [1]. It encodes the deadenylase NOCTURNIN, which is implicated in a number of metabolic processes through posttranscriptional circadian regulation of genes involved in metabolism [2, 3]. In mice, NOCTURNIN is the sole deadenylase affecting the high-amplitude rhythms and shows nocturnal peaks, whereas most other deadenylases are arrhythmic or have very low-amplitude rhythms that peak during the day [2]. Several metabolic pathways are related to the circadian clock [4]. The* NOCTURNIN* gene regulates the circadian cycles and binds them to the metabolic rhythms; its involvement in lipid metabolism, adipogenesis, glycaemic homeostasis, and inflammation entails that it plays a role in promoting metabolic alterations [5–7].* Nocturnin-*knockout mice show greater glucose tolerance and insulin sensitivity as well as increased resistance to obesity and hepatic steatosis, likely due to altered absorption of intestinal fats or to their consumption in alternative metabolic cycles [3]. The latter findings underscore the important role of the gene in adiposity regulation. A recent study of* NOCTURNIN* expression in subcutaneous and visceral adipose tissue from obese and nonobese Chinese subjects has found higher levels in obese individuals in both adipose depots [8].

Circadian desynchronization due to shift work affects not only the central nervous system, by altering the wake/sleep and hunger/satiety cycles [9], but also peripheral tissues, where clock-controlled genes are functionally regulated by the suprachiasmatic nucleus (SCN) and in turn regulate metabolic processes [10–12]. The consequences of circadian rhythm desynchronization on the health and wellbeing of shift workers are mainly related to shift frequency, duration, regularity, and type of rotation [13–15]. Shift and night work increases the risk of developing chronic conditions such as ischaemic heart disease [16–18], type 2 diabetes [18–21], metabolic syndrome [22, 23], and cancer [15, 24]. Indeed, overweight and obesity are more frequent among shift workers than among daytime workers [25–28]. The trigger for the development of further metabolic disorders is abdominal adiposity [26, 27, 29]. The causes of this association are still unclear.

The aim of this study was to investigate the diurnal* NOCTURNIN* variation in healthy subjects and to test for differences in its expression levels in shift and daytime workers.

## 2. Materials and Methods

### 2.1. Diurnal Variation of NOCTURNIN Expression in Healthy Volunteers

*NOCTURNIN* is a clock-controlled gene which in rodents shows a variable expression during the day. To establish whether such variation also occurs in humans, its expression levels were investigated in lymphocytes from 15 healthy subjects, 8 men and 7 women aged 27-39 years [mean±standard deviation (SD), 32.4±4.3 years], who had been enrolled in a previous study [30]. The volunteers filled out a questionnaire to provide personal data and information on the inclusion criteria, thereby also providing their informed consent. The study was carried out according to the principles of the Declaration of Helsinki. Samples were processed after approval of a written consent statement (Prot. No. 737) by the Ethics Committee of Catania (Italy).

The inclusion criteria comprised regular sleep/wake patterns and a negative history of severe physical diseases; participants were excluded if they had been on medications or had made journeys across time zones in the previous two months. The volunteers' health status was established by physical examination. All subjects agreed to follow their daily routines and to sleep 8 hours at regular times in the dark for 7 days before testing.

Testing took place in the laboratory from 8:00 AM of one day to 8:00 AM the next day. In the 24-hour interval, subjects were allowed to move and drink ad libitum from 8:00 AM to 12:00 AM (awake time) and slept in the same room from 12:00 AM to 8:00 AM (sleep time). Breakfast was at 8:30 AM, lunch at 12:30 PM, and dinner at 8:30 PM. The light intensity in the laboratory was measured at eye level using a Minolta Chroma Meter CL-100 (Minolta Camera Company Ltd., Osaka, Japan). Its mean intensity during wake time was 407.7±112.5 lux, provided by 4000° K fluorescent lamps (Osram Lumilux, Osram, Münich, Germany) and 2.6±2.2 lux during sleep time, provided by a bulb emitting red (700° K) light (Philips PAR38 IR, Philips Lighting, Eindhoven, The Netherlands). Room temperature was 22±1°C throughout the study. Blood was collected in EDTA glass tubes every 4 h (8:00 AM, 12:00 PM, 4:00 PM, 8:00 PM, 12:00 AM, 4:00 AM, and 8:00 AM); lymphocytes were obtained immediately afterwards.

### 2.2. NOCTURNIN Gene Expression in Shift Workers

Forty nurses were selected among the staff of Area Vasta 2 (Ancona, Italy) as they underwent the periodic medical examinations envisaged by Italian regulations for health personnel; of these, 20 were involved in fast clockwise shift rotation that included night shifts (SW nurses), whereas 20 worked exclusively daytime shifts (DT nurses). All nurses were female.

The study criteria required participants to

- be taking no drugs, melatonin supplements, or oestrogen/progestin treatment
- suffer from no psychiatric disorders, degenerative or cardiovascular disease (e.g. heart attack and stroke), insomnia, chronic viral infections, cancer, autoimmune disorders, or diseases such as uterine fibromatosis and polycystic ovary syndrome
- have worked the same shift for at least 2 years without changes (e.g., by swapping shifts with colleagues) for at least 6 months prior to testing
- work at least 48 night shifts a year (SW nurses)
- have no occupational exposure to ionizing radiation or antiblastic drug preparation

Nurses provided their consent after receiving information about the aims and modalities of the study, which was conducted according to the Helsinki Statement of Ethical Standards. Being an integral part of the routine health surveillance, the study was not subject to formal approval by the local ethics committee. The schedule of SW nurses was as follows: day 1, 7:00 AM–2:00 PM; day 2, 2:00 PM–10:00 PM; day 3, 10:00 PM–07:00 AM; rest for 48 h; resumption of the cycle. The DT nurses worked a 07:00 AM–2:00 PM shift 6 days a week. To avoid the acute alterations associated with a night shift (SW nurses), all participants were tested during a day shift after a regular night sleep.

At 8:00 AM, venous blood was collected to evaluate fasting glycaemia, glycated haemoglobin, and lipids (total cholesterol, HDL cholesterol, LDL cholesterol, and triglycerides) and to obtain lymphocytes for measuring* NOCTURNIN* expression levels.

Subsequently, participants underwent medical examination and measurement of anthropometric parameters including body weight, height, blood pressure, body mass index (BMI), waist circumference, hip circumference, and skinfold (biceps, triceps, subscapular, and suprailiac) thickness. The Durnin and Womersley formula were used for fat mass calculation [31]. General information, including smoking habits and alcohol consumption, was collected as part of the medical history. Participants were asked to keep a food diary, reporting daily food intake and amount and time of intake for 5 consecutive days. A dietician explained how to compile it. Total energy intake and fat, carbohydrate, and protein intake were calculated [32] and expressed as daily average for each of the 5 days.

During physical examination, a SenseWear Armband® (SWA) (BodyMedia Inc., Pittsburgh, PA, USA), a metabolic monitor that quantifies energy expenditure using internal sensors, was secured high on each participant's right arm and activated. The device consists of a two-axis accelerometer that measures movement through a microelectromechanical sensor, a galvanic sensor that records skin responses, a skin temperature sensor, and a sensor measuring external temperature [33]. The SWA was worn for 5 days. Total energy expenditure, energy expenditure due to motor activity, duration of physical activity (> 3.0 Metabolic Equivalents, METs), time lying down, and sleep duration were calculated using the SenseWear Armband® Software. There were no cases of interrupted or invalid recordings. Results were expressed as daily average for each of the 5 days.

### 2.3. NOCTURNIN Gene Expression Analysis

The peripheral blood samples were processed immediately after collection. Lymphocytes were isolated using a density gradient separation medium (Cedarlane Laboratories LTD, Hornby, ON, Canada) and stored at −80°C until RNA extraction. Total RNA was isolated using RNeasy Mini Kit (Qiagen, Hilden, Germany) according to the manufacturer's instructions. RNA quality and quantity were evaluated with a Nanodrop 1000 spectrophotometer (Thermo Scientific, Wilmington, DE, USA). cDNA was synthesized according to the High-Capacity cDNA Reverse Transcription Kit protocol (Applied Biosystems, Foster City, CA, USA).

Gene expression was analysed by real-time quantitative PCR (RT-qPCR) using TaqMan Gene Expression Master Mix (Applied Biosystems). To control for variations in the amount of cDNA available for RT-qPCR in the different samples, the gene expression levels of the target sequences were normalized to the expression of an endogenous control, glyceraldehyde-3-phosphate dehydrogenase (*GAPDH*). The primer and probe sets used for RT-qPCR assays were as follows: nm012118.1.pt.CCRN4L for* NOCTURNIN* and Hs.PT.39a.22214836 for* GAPDH* (Integrated DNA Technologies Inc., Coralville, IA, USA).* NOCTURNIN* levels were calculated by the equation: 2^−∆Ct^ [34].

### 2.4. Statistical Analysis

A total sample size of 10 healthy volunteers was calculated a priori, to detect significant differences in* NOCTURNIN* expression with an effect size of 0.60, a power ≥ 0.80, and an *α*=0.05 (two-tailed). The theoretical sample size was increased by 50% to achieve more representative data. Normality of distribution was assessed with the Kolmogorov-Smirnov test. Since the transformed values approximated most closely a normal distribution, natural logarithms of* NOCTURNIN* values were used for the analysis. Continuous variables were expressed as mean ± SD and log-transformed variables as geometric mean and 95% confidence interval (95% CI). One-way repeated measures ANOVA was run to analyse* NOCTURNIN* expression at different time points. Mauchly's test was performed to verify the sphericity assumption; an LSD test was used as a post hoc assessment. Cosinor analysis was applied to study circadian rhythmicity. Student's* t*-test was used to test differences in independent measures between SW and DT nurses. The chi-square test was applied to test dichotomous parameters. Multivariate linear regression analysis was used to assess the dependency of* NOCTURNIN* expression levels on shift work. Explanatory variables associated with the outcome at a significance level of ≤ 0.20 (univariate analysis) were included as independent variables. BMI, total energy expenditure, and total food intake were considered as potential confounders a priori. Data were analysed with SPSS software (SPSS, Chicago, IL, USA) and Circadian software (available online at www.circadian.org). Statistical significance was set at p < 0.05.

## 3. Results

**Figure 1:**
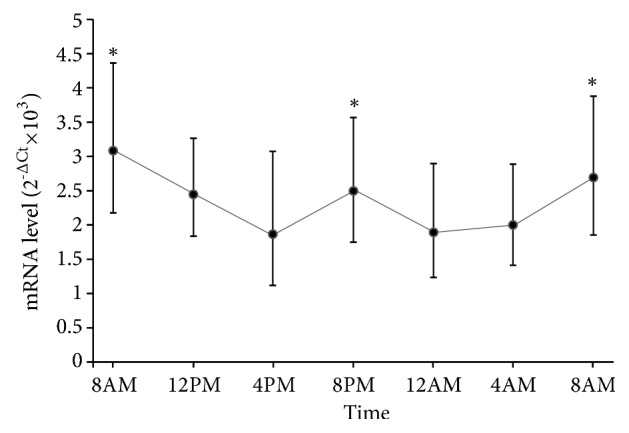
*NOCTURNIN* expression profiles calculated in lymphocytes from 15 healthy volunteers collected at 4-hour intervals over 24 h.* NOCTURNIN* mRNA levels are expressed as geometric mean ± 95% confidence interval. A significant diurnal variation was highlighted by repeated measures ANOVA and Cosinor analysis.*∗* p < 0.05 compared to nearest times.

Analysis of the* NOCTURNIN* mRNA expression pattern in lymphocytes from the 15 healthy volunteers showed a significant diurnal variation (repeated measures ANOVA; p < 0.05; Figure 1) with the main peak at 8:00 AM and a secondary peak at 8:00 PM (repeated measures ANOVA, post hoc LSD, p < 0.05). There were no significant differences between men and women. Cosinor analysis confirmed the ANOVA results and yielded a period of 12.2 h, a mesor of 2.49, an amplitude of 0.49, and an acrophase of -22° for the oscillation of* NOCTURNIN* expression.

All 40 nurses involved in the study were female. Both groups consisted of participants coming from several different departments. SW nurses were significantly younger than DT nurses (p=0.027) and had significantly less job seniority (p=0.002) (Table 1). Shift work seniority for SW nurses was 19.9±4.2 years and coincided with job seniority, demonstrating that SW nurses had not changed their schedules for at least 2 years prior to the study. They worked 6.0±1.0 nights per month. There were no significant differences between SW and DT nurses in the number of smokers or of alcohol drinkers.

There were no significant differences in BMI, waist or hip circumference, body fat, or systolic/diastolic pressure between SW and DT nurses. Similarly, fasting glycaemia, glycated haemoglobin, total cholesterol, HDL cholesterol, LDL cholesterol, and triglycerides did not show significant differences between the groups (Table 2).

The analysis of total energy intake and diet composition did not show significant differences between SW and DT nurses (Table 3).

Total energy expenditure and energy expenditure due to motor activity, as calculated by the SWA software, were not significantly different between the groups (Table 4). However, SW nurses spent several more minutes performing intense physical activities (p=0.029) as well as more time lying down (p=0.041) than DT nurses. Sleep duration was not significantly different.

**Figure 2:**
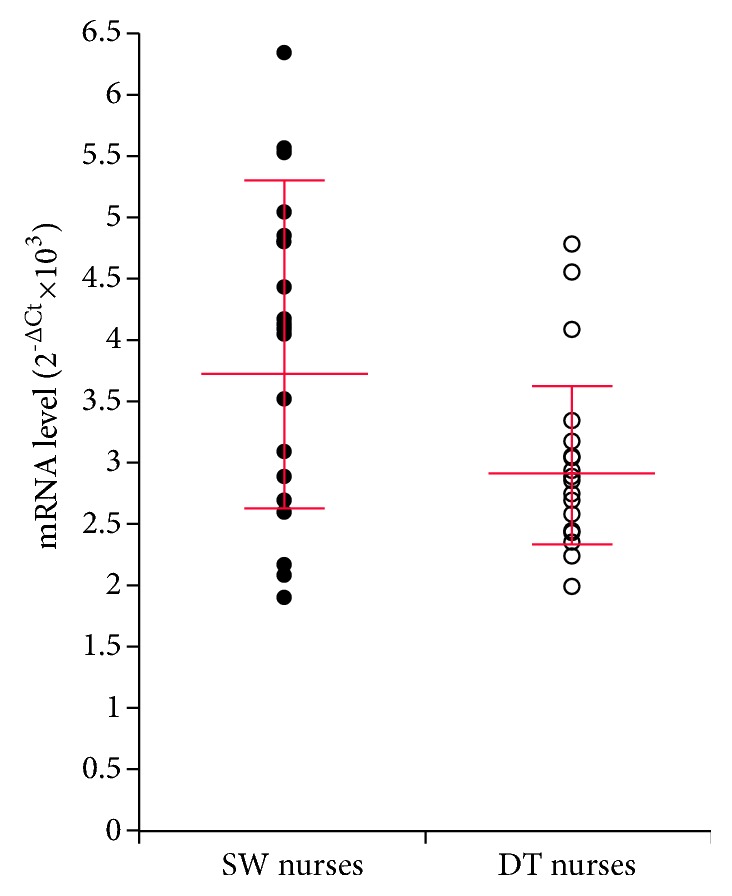
*NOCTURNIN* expression levels in lymphocytes from shift-working (SW) and daytime (DT) nurses sampled at 8:00 AM. Red lines: geometric mean ±95% confidence intervals.

*NOCTURNIN* expression levels, measured in peripheral venous blood lymphocytes sampled at 8:00 AM, were significantly higher in SW than in DT nurses [GM (95% CI) 3.74 (2.64-5.31) 2^−∆Ct^x10^3^ vs. 2.92 (2.34-3.63) 2^−∆Ct^x10^3^, respectively; p=0.010] (Figure 2).

Multivariate analysis confirmed a positive dependency of* NOCTURNIN* expression levels on shift work (*β* 0.533 p=0.009). It also highlighted a positive correlation of* NOCTURNIN* levels with BMI (*β* 0.770 p=0.002) and a negative correlation with total energy expenditure (*β* -0.470 p=0.036) (Table 5).

## 4. Discussion

NOCTURNIN is the only deadenylase showing high-amplitude rhythms in mice [2]. Tests in human volunteers showed diurnal variation in* NOCTURNIN* gene expression, with the main peak at 8:00 AM and a secondary peak at 8:00 PM. In mice, the peak corresponds with the peak of the* PER2* gene [35]. Since human* PER2* expression peaks in the early morning [30, 36], the main* NOCTURNIN* peak measured at 8:00 AM in the present study was expected.

Various intestinal functions such as peristalsis, emptying, gastric pH, and absorption of food are ultradian events [37]. The secondary* NOCTURNIN* peak may be related to food intake and intestinal activities [38].

The* NOCTURNIN* gene links the circadian cycles to the metabolic rhythms and, given its specific role in fat absorption and metabolism [37, 39, 40], it may be implicated in promoting metabolic alterations [5–7]. Shift work has been shown to be associated with metabolic alterations and obesity, although the causes of the association have not been fully clarified. The hypothesis that shift workers would eat more calories and/or fatty foods has not been demonstrated conclusively [41–44]. There are also conflicting data regarding energy expenditure and physical activity as assessed by self-administered questionnaires [45–47]. However, sleep deprivation, which is common among shift workers, is accompanied by weight gain [26, 27] and by increased ghrelin and reduced leptin concentrations in the circulation [48, 49]. An imbalance due to increased food intake and reduced energy expenditure may be the cause of the weight gain and metabolic alterations reported in shift workers. Our findings indicate that total energy expenditure, energy expenditure due to motor activity, and sleep duration were not significantly different between SW and DT nurses. The longer time spent by SW nurses performing intense physical activity was probably compensated for by the longer time spent by SW nurses lying down. The food diaries also failed to demonstrate significant differences in energy intake or diet composition between the two groups. The same applies to their metabolic parameters.* Nocturnin-*knockout mice are resistant to diet-induced obesity. This is not due to increased physical activity, reduced food intake, or accelerated metabolism, but probably to a metabolic deficit or reduced lipid absorption [3].

*NOCTURNIN* expression was significantly higher in SW nurses, and multivariate analysis confirmed the role of shift work as an independent factor influencing* NOCTURNIN* expression. Circadian desynchronization associated with shift work involves both the SCN and the expression of peripheral clock genes [36, 50] which, in turn, tune the expression of clock-controlled genes such as* NOCTURNIN*. Chronic* NOCTURNIN* overexpression may promote metabolic disorders through increased absorption and storage of fats in adipocytes. In addition,* NOCTURNIN* seems to be involved in the regulation of glucose homeostasis and insulin sensitivity and may promote insulin resistance [2]. It has also been reported that its expression may be influenced by hormones like melatonin and cortisol, whose rhythms are affected by the circadian disruption induced by shift work [36, 51, 52]. Melatonin is a regulator of fat metabolism, and its predominantly antiadipogenic activity suggests that it may be a* NOCTURNIN* antagonist [53]. Although no information is available on the interaction between* NOCTURNIN* and cortisol, antagonism may be hypothesized in this case, too.

The selection of* GAPDH* as the reference gene was due to its successful use in* NOCTURNIN* mRNA studies [54] and in investigations of clock gene expression in human tissues [30, 55, 56]. Notably, some studies have reported that* GAPDH* is rhythmically expressed in some mice and rat tissues [57–60]. Although no data are available about the rhythmicity of* GAPDH* expression in human blood lymphocytes, it has been reported that the evidence for gene rhythmicity can be impaired by using* GAPDH* as the reference gene [58]. Although we have found diurnal variation in* NOCTURNIN* gene expression, the use of* GAPDH* as the reference gene may be a limitation of the study.

The significantly different* NOCTURNIN* gene expression levels found in SW and DT nurses related to the specific type of shifts examined. Notably, blood was sampled after a day off and a regular night sleep, to avoid the acute alterations induced by night work. Data from workers with a shift schedule that includes several consecutive nights would provide valuable information, since a larger effect might be expected. The values obtained in the present study are also affected by the time of blood collection. The single sample taken from the nurses clearly prevented examination of diurnal variation. However, the peak hour is probably the best time to investigate difference in* NOCTURNIN* expression levels between SW and DT nurses. The interindividual variability in* NOCTURNIN* gene expression was greater among SW nurses, a finding that probably depends on a different tolerance of shift work. Although several factors influence the response to shift work, chronotype probably plays a major role [61–64]. BMI and waist circumference, which are usually significantly different in shift compared with daytime workers [22, 27, 29, 65], did not reach significance in the present study, suggesting that the effect size of shift work on these parameters is lower than the effect on* NOCTURNIN* expression levels. However, multivariate analysis highlighted a positive correlation between* NOCTURNIN* expression and BMI, in line with a previous study [8]. Moreover, the inverse correlation found between* NOCTURNIN* expression and total energy expenditure suggests that shift workers should be encouraged to engage in physical activity.

## 5. Conclusions

The human* NOCTURNIN* gene is a clock-controlled gene characterized by diurnal variation and a peak at 8:00 AM. Measurement of its expression level in peripheral blood lymphocytes at 8:00 AM can provide useful information on the relationship between changes in circadian rhythm and metabolic disorders. It may be hypothesized that the higher* NOCTURNIN* expression levels found in shift workers in this study have the potential to promote metabolic alterations and/or activation of alternative metabolic pathways that may increase the risk of developing obesity and metabolic syndrome.

## Figures and Tables

**Table 1:** Demographic characteristics of shift-working (SW) and daytime (DT) nurses [SD= standard deviation].

	SW nurses			DT nurses			P-value
	(n=20)			(n=20)			
	Mean	SD	%	Mean	SD	%	
Age (years)	43.6	3.2		45.9	3.1		0.027
Job seniority (years)	19.9	4.2		24.5	4.5		0.002
Shift work seniority (years)	19.9	4.2					
Nights worked per month	6.0	1.0					
Smokers (%)			35.0			50.0	0.337
Alcohol drinkers (%)			25.0			35.0	0.490

**Table 2:** Metabolic characteristics and blood pressure of shift-working (SW) and daytime (DT) nurses [SD= standard deviation].

	SW nurses		DT nurses		P-value
	(n=20)		(n=20)		
	Mean	SD	Mean	SD	
BMI	30.0	8.4	27.4	5.3	0.249
Waist circumference (cm)	86.3	17.0	83.1	10.2	0.475
Hip circumference (cm)	97.2	14.9	93.7	9.5	0.381
Body fat (%)	37.7	5.1	37.5	4.8	0.899
Systolic pressure (mmHg)	120.3	13.2	117.9	10.8	0.533
Diastolic pressure (mmHg)	78.0	8.5	75.4	6.8	0.292
Fasting glycaemia (mg/dl)	87.0	9.5	89.7	15.1	0.503
Glycated haemoglobin (%)	5.2	0.3	5.3	0.2	0.223
Total cholesterol (mg/dl)	194.5	34.1	210.0	44.1	0.221
HDL cholesterol (mg/dl)	52.0	9.9	57.3	16.9	0.234
LDL cholesterol (mg/dl)	127.5	30.6	133.9	36.5	0.552
Triglycerides (mg/dl)	114.5	75.1	92.1	46.7	0.264

**Table 3:** Energy intake and diet composition in shift-working (SW) and daytime (DT) nurses [SD= standard deviation].

	SW nurses		DT nurses		P-value
	(n=20)		(n=20)		
	Mean	SD	Mean	SD	
Total energy intake (kcal)	2128	305	2030	376	0.371
Fat (%)	25.9	0.8	26.3	0.9	0.146
Carbohydrates (%)	54.7	1.7	54.5	2.3	0.756
Protein (%)	19.3	0.9	19.2	1.1	0.877

**Table 4:** Energy expenditure in shift-working (SW) and daytime (DT) nurses [SD= standard deviation].

	SW nurses		DT nurses		P-value
	(n=20)		(n=20)		
	Mean	SD	Mean	SD	
Total energy expenditure (kcal)	2010	687	1955	708	0.805
Energy expenditure due to motor activity (kcal)	1332	531	1277	567	0.753
Physical activity (minutes)^a^	144	111	82	67	0.039
Time lying down (minutes)	495	62	457	51	0.041
Sleep duration (minutes)	403	72	382	56	0.310

^a^physical activity > 3 METs.

**Table 5:** Effect of shift work, age, BMI, total energy expenditure, physical activity, time lying down, and total food intake on *NOCTURNIN* expression levels. Results of multivariate analysis.

	*NOCTURNIN*	
	gene expression	
	*β*	P-value
Shift work	0.533	0.009
Age	0.147	0.417
BMI	0.770	0.002
Total energy intake	-0.098	0.403
Total energy expenditure	-0.470	0.036
Physical activity^a^	-0.056	0.788
Time lying down	-0.138	0.410

^a^physical activity >3 METs.

## Data Availability

The data used to support the findings of this study are included within the article.

## Supplementary Materials

Supplementary Materials The supplementary file includes individual profiles of* NOCTURNIN* expression of healthy volunteers. There were no significant differences between men and women.
