# Supplementary figures and images for: NOCTURNIN Gene Diurnal Variation in Healthy Volunteers and Expression Levels in Shift Workers
Source: Biomed Res Int. 2019 Jul 31;2019:7582734. doi: 10.1155/2019/7582734 (PMC6699378; doi:10.1155/2019/7582734)

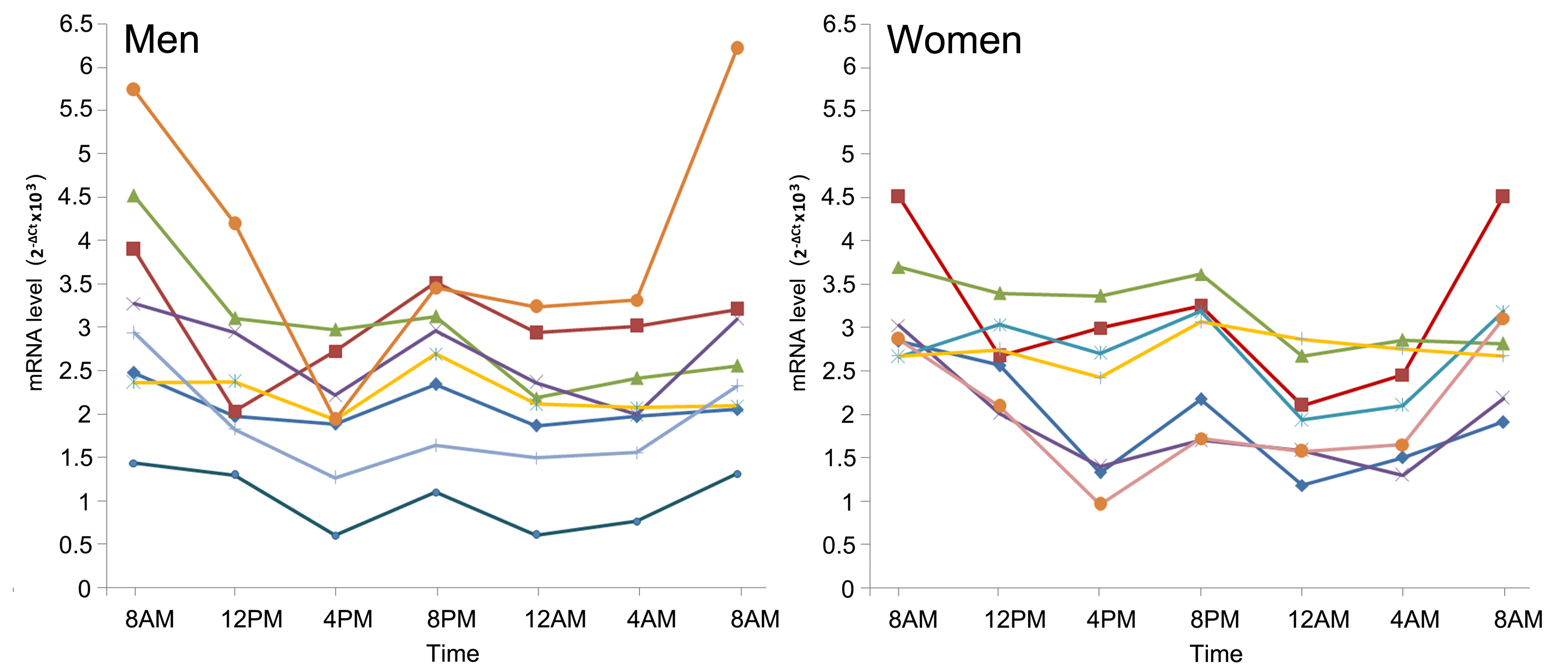

Supplement: Supplementary Materials — The supplementary file includes individual profiles of NOCTURNIN expression of healthy volunteers. There were no significant differences between men and women. [file 7582734.f1.jpg]
